# In search of the perfect tan: Chemical activity, biological effects, business considerations, and consumer implications of dihydroxyacetone sunless tanning products

**DOI:** 10.1111/jocd.14968

**Published:** 2022-04-21

**Authors:** Josh Turner, Danielle A. O’Loughlin, Phill Green, Tom O. McDonald, Kevin J. Hamill

**Affiliations:** ^1^ 4591 Institute of Life Course and Medical Sciences University of Liverpool Liverpool UK; ^2^ PZ Cussons Manufacturing Manchester UK; ^3^ 4591 Department of Chemistry University of Liverpool Liverpool UK

**Keywords:** artificial tanning, self‐tanning products, skin

## Abstract

As the desire and popularity of a tanned appearance continues, the social effects of UV‐free tanning are becoming more important. Dihydroxyacetone (DHA) has seen extensive use as the main tanning agent in sunless tanners. The DHA‐induced tan is a result of brown melanoidins formed by a non‐enzymatic Maillard reaction between DHA and amino acid species found in the stratum corneum. DHA, thereby, provides a safer route to a tanned appearance compared with exposure to ultraviolet radiation. However, DHA is a highly reactive molecule, posing a multitude of challenges for potential product formulations. With their increased use, the safety considerations of topically applied DHA tanners have been investigated. Many different vehicles have been used for topical delivery of DHA, and they are becoming increasingly multifunctional. This review provides a holistic overview of dihydroxyacetone sunless tanning products.

## 1 CONTEXT & STRUCTURE

The tanning effect of dihydroxyacetone (DHA) was first reported by Eva Wittgenstein, as a side‐effect in children taking DHA solutions for glycogen storage disease, where those that had spilled their DHA solutions exhibited darkening of the skin that had contacted the DHA solution.^1^, ^2^, ^3^ Throughout the rest of the 20^th^ century, tanning products saw some use but were plagued by poor product performance such as undesirable color tones and streakiness. In the 21^st^ century, the research into DHA and therefore the understanding of the complex reactions involved in sunless tanning has increased dramatically. DHA‐containing sunless tanners have improved in performance through formulation advancements and seen increased popularity. Recent articles have discussed the safety and health considerations of DHA sunless tanners and we encourage readers to refer to these reviews.^4^, ^5^, ^6^ Here, we present a holistic and current review of DHA sunless tanning.

First, we discuss the fundamental role of DHA in sunless tanning products; the chemistry of DHA and proposed reaction pathways for melanoidin formation, the structure and function of the stratum corneum biological substrate for the tanning reaction, and comparison with UV exposure tanning. Next, the topic of DHA sunless tanning products is considered from the perspective of business and industry; why they are a marketable product, formulation considerations, and limitations of DHA sunless tanning products, and the different product types currently available for DHA sunless tanners. The final section discusses the social implications of sunless tanning, advice for the industry and consumers, and the potential health benefits and risks associated with sunless tanners.

## 2 BACKGROUND INFORMATION

### 2.1 Dihydroxyacetone and Maillard reaction chemistries

**FIGURE 1:**
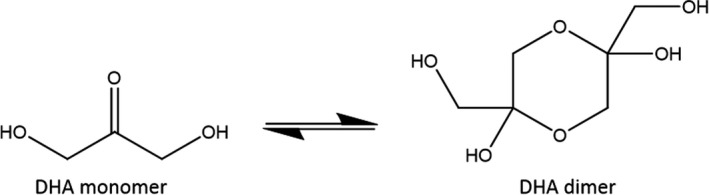
Scheme showing the structure of DHA in equilibrium with its monomeric and dimeric forms. The dimeric form is favored when DHA is a solid, whereas the monomer form is typically found in solution

Dihydroxyacetone, also known as glycerone or 1,3‐dihydroxypropan‐2‐one, is the only ketotriose monosaccharide. It has the chemical formula of C_3_H_6_O_3_, which it shares with its structural isomers L‐glyceraldehyde and D‐glyceraldehyde. DHA can be found naturally in the human body and, in its phosphorylated form, plays an important biological function in the glycolysis metabolic pathway.^7^ Commercial DHA is typically produced by microbial fermentation of glycerol using *Gluconobacter oxydans*, although other methods of production such as electrocatalytic oxidation have been investigated to meet the sunless tanner‐driven increase in demand.^8^ Pure DHA exists as a white, crystalline powder with hygroscopic properties under ambient conditions and is generally dimeric as a solid, reverting to monomeric in solution (Figure 1).

For many markets, DHA is synonymous with sunless tanning. For example, in the United States DHA is the only sunless tanning agent the Food and Drug Administration (FDA) allows,^9^ although some other additives such as D‐erythrulose may be used in addition to DHA. When a sunless tanner is applied topically to the skin, the DHA penetrates into the outermost layer of the skin and undergoes what is generally considered a Maillard or Maillard‐like reaction.^9^, ^10^, ^11^ The Maillard reaction forms multiple products, many of which are heterogeneous, brown, nitrogenous pigments collectively known as melanoidins.^12^ It is the melanoidins that result in the tan appearance after sunless tanning product application. However, there is debate as to whether DHA undergoes a true Maillard reaction when it is topically applied to skin^13^; this will be discussed after first presenting the Maillard reaction.

**FIGURE 2:**
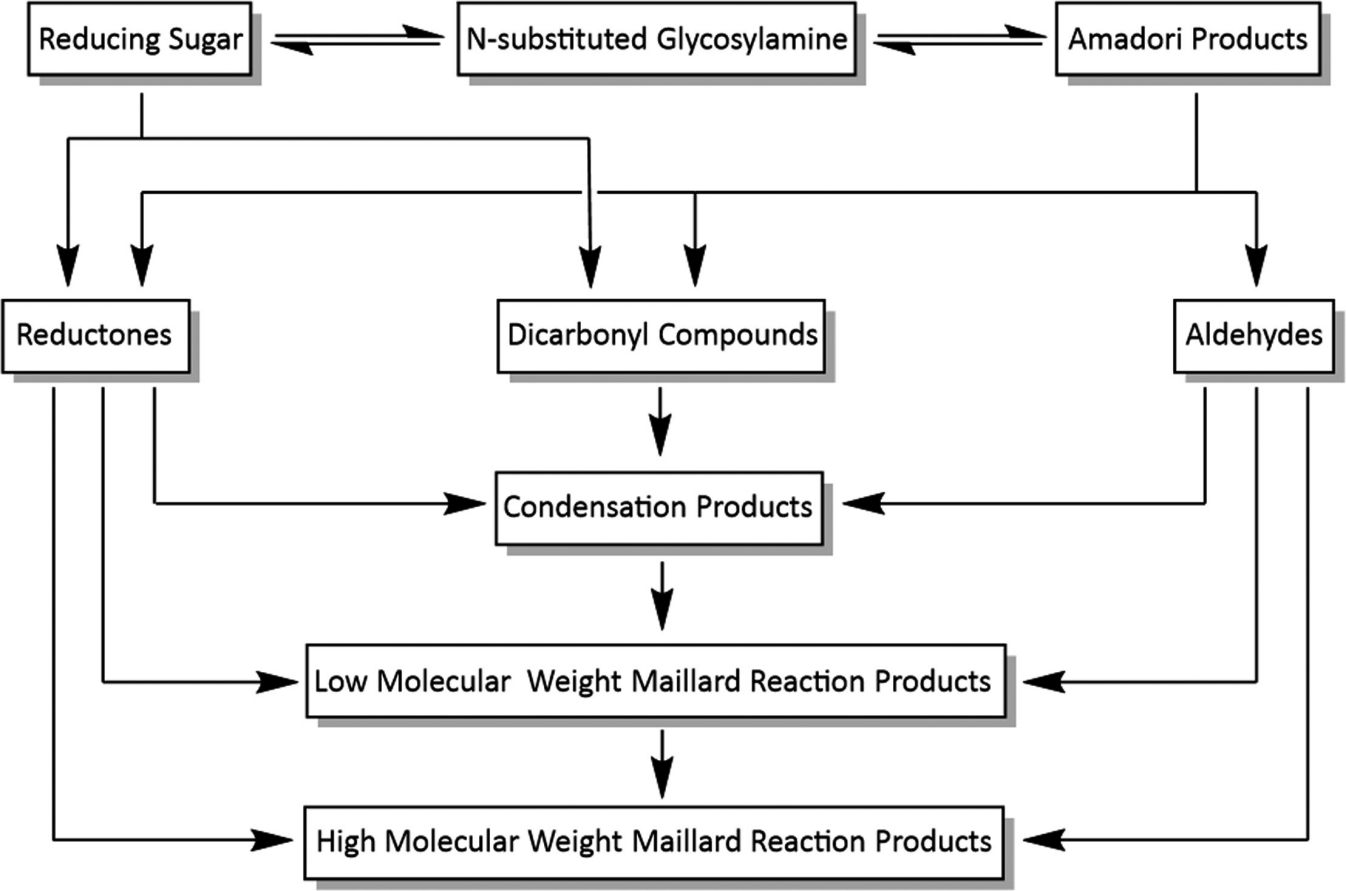
General overview of the Maillard reaction pathway, highlighting the rising complexity through the cascade

Many valuable lessons about the Maillard reaction have been learned from food science research, where this reaction is often associated with non‐enzymatic browning. There are many steps to the Maillard reaction (Figure 2), although the full mechanism is yet to be firmly established and it should be noted that in the food science context, the experiments often involve mixing reducing sugars and proteins under the elevated temperatures associated with cooking. It is now generally accepted that the reaction proceeds through several intermediates, it can be acid‐ or base‐catalyzed, and ultimately generates an extremely complex mixture of reaction products. These Maillard reaction products are often categorized as either high molecular weight or low molecular weight, although what constitutes these actual weights varies between authors.^12^ High molecular weight may be >5 kDa,^14^ >12 kDa,^15^, ^16^ or even >60 kDa,^17^ whereas low molecular weight may be <3.5 kDa,.^18^, ^19^ The tanning‐associated melanoidin brown pigments belong to the high and possibly low molecular weight fractions of Maillard reaction products.^12^

**FIGURE 3:**
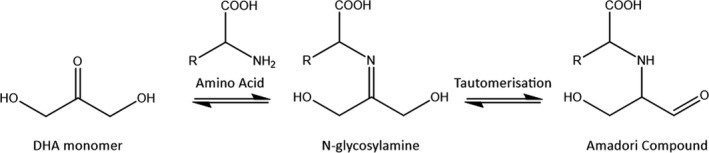
Scheme showing the initial steps of the Maillard reaction between DHA and a generic amino acid, including the formation of the N‐glycosylamine intermediate and subsequent tautomerization to an Amadori compound

Despite the overall complexity of the Maillard reaction, the initial reactive pathway is relatively well understood (Figure 3). A condensation reaction between a reducing sugar and an amino acid results in the formation of an N‐substituted glycosylamine, via a hemiaminal intermediate. This is followed by Amadori rearrangement, or tautomerization, to form the intermediate Amadori compounds (Figure 3). From here it is believed that a complex cascade of dehydration, deamination, Strecker degradation, fragmentation, and polymerization reactions occur between the various reactants and intermediates, resulting in a large distribution of products. In one paper, the formation of over 300 unique species was reported after 10 h of Maillard reaction between just two reactants.^20^ Another report found that several thousand different chemical species were formed under several different Maillard reaction conditions, with different reactivities being displayed by different free amino acids.^21^

For the Maillard reaction in sunless tanning, the reducing sugar is DHA while the other reactants are typically free amino acids in the stratum corneum (Figure 3). Although a single structure is not defined for a melanoidin, it is generally agreed that they are a class of large, brown, nitrogenous polymers that are formed during the late stages of the Maillard reaction. Melanoidins are likely to consist of a large, conjugated electronic structure similar to melanin, the pigment found in human skin. It is not currently understood whether melanoidins are large structures themselves, or whether they are smaller chromophoric motifs that are integrated into the larger high molecular weight Maillard reaction products during polymerization reactions. It has been suggested that there are three potential routes for melanoidin formation: the polymerization of low molecular weight Maillard reaction products, cross‐linking of low molecular weight chromophores and amino acid side chains, or sugar degradation products branched with amino acids.^12^

As mentioned previously, there is some debate as to the true nature of the reaction between DHA and skin. Johnson and Fusaro present compelling evidence that DHA bypasses many of the early steps of the Maillard reaction,^13^ such as those presented at the top of Figure 2 and in Figure 3. The use of various DHA analogs allowed the authors to deduce that two hydroxyl groups adjacent to a carbonyl moiety were required for browning to occur in their test model. It is also proposed that oxidative amination is unlikely to be involved early in the reaction pathway. Enolization, carbonyl migration, fission, and Strecker degradation are also reported to be absent or of limited importance. The authors instead propose that a pathway involving unreacted DHA is most likely, due to the maximum attainable browning varying directly with DHA concentration. The later steps of the Maillard reaction are still believed to take place, resulting in a complex mixture of large, brown, nitrogenous polymers that can still be referred to as melanoidins.

### 2.2 The stratum corneum

The stratum corneum (SC) is the outermost layer of human skin, and is the site of action for DHA. The SC is the interface between the human body and the outside world. It provides both a physical barrier to prevent the entry of unwanted materials into the body, and reduces water loss. The SC is typically referred to as the “dead” layer of surface skin, yet this is not completely true. Although the cells of the SC (corneocytes) are nondividing, the extracellular matrix is metabolically active.^22^

**FIGURE 4:**
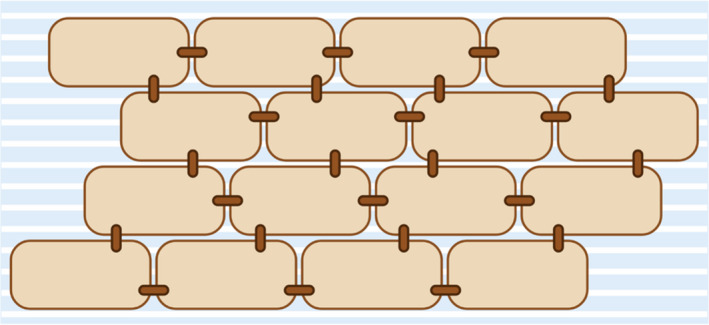
Simple bricks and mortar interpretation of the stratum corneum, with corneocytes as bricks, extracellular lipid matrix as the blue and white mortar, and the corneodesmosomes as brown rivets. Created with BioRender.com

A simplified model of the structure can be achieved by likening it to a bricks and mortar; the corneocyte cells acting as the bricks, and the intercellular lipid matrix the mortar (Figure 4). The physical barrier function is mainly performed by the corneocytes, while the water permeability barrier is achieved by the lipid matrix. The corneocytes are typically stacked in layers of ca. 20 cells thick, although this varies largely depending on the anatomical location.^23^ As the skin is constantly being regenerated, the corneocytes are shed from the surface in a process called desquamation; it is this process that causes a sunless tan to fade and disappear over a course of several days. As with the thickness of the SC, rates of desquamation vary with anatomical location.

The structure and function of the skin present challenges to the sunless tanning industry. Inherently, the function of the SC is to behave as a barrier and yet the tanning response relies on the penetration of DHA into the SC. This often necessitates the use of penetration enhancers. Moreover, as the thickness of the SC and rate of desquamation vary with anatomical location, obtaining an even and consistent tan is challenging for the tanning product formulator. Additionally, interpersonal differences in terms of composition and natural pigmentation of skin complicate things further still.

Unsurprisingly, the SC is more complex than simply cells and lipids; several other important aspects define its function and behavior and may influence the DHA reaction. These include corneodesmosomes, protein‐based linkages between corneocytes which physically hold the “bricks” together; lipolytic and proteolytic enzymes which process the material released from dead and dying cells; and lamellar body secretions. In addition, the intercellular lipid matrix is composed of many different species of ceramides, cholesterol, fatty acids, and cholesterol and glucosylceramide derivatives. It is unclear if or how these additional components of the SC affect the reaction of DHA.

Another important property of the SC is its acidic pH. Moreover, and, importantly for considering product development, there is a large range of pHs observed between 4 and 6.^22^, ^24^, ^25^ The acidic pH is controlled and maintained at the acid mantle, a thin acidic film at the surface of the SC consisting of sebum, sweat, lactic acid, urocanic acid, fatty acids, and pyrrolidine carboxylic acid.^26^, ^27^ The acidic pH facilitates enzymatic activities, limits cellular water loss, acts as defense against harmful foreign bodies such as bacteria, and maintains the integrity and adhesion of the SC. The actual pH of the skin is affected by many intrinsic and extrinsic factors, such as anatomical location, age, race, gender, and use of personal hygiene and cosmetic products.^28^, ^29^ As such, the pH of a topically applied cosmetic such as a sunless tanning product must be carefully considered so as to not disrupt the pH balance of the SC.

**FIGURE 5:**
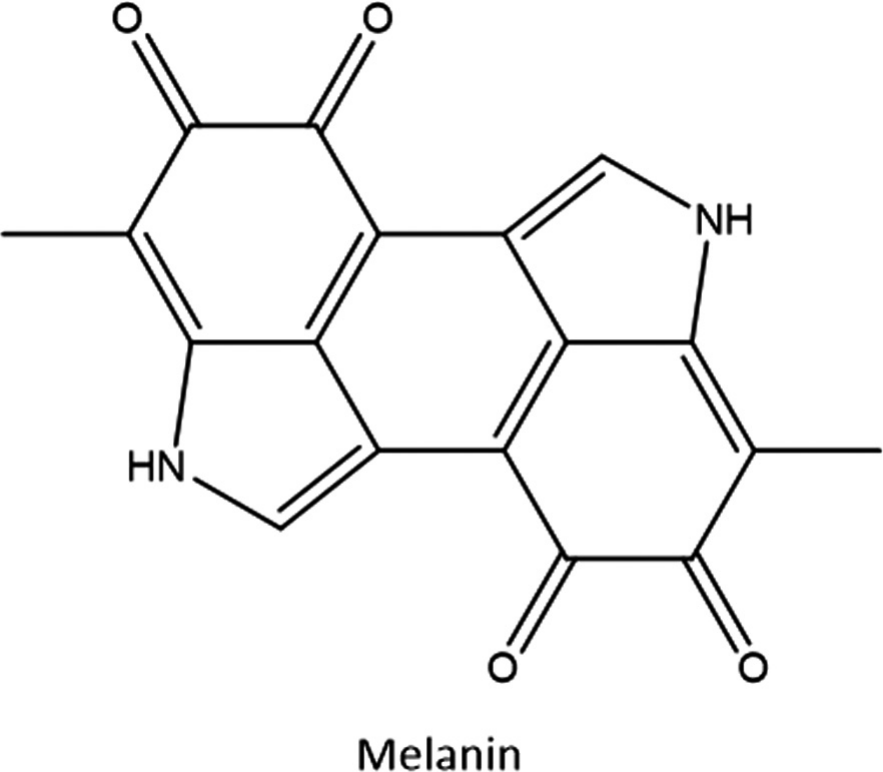
Structure of melanin

**FIGURE 6:**
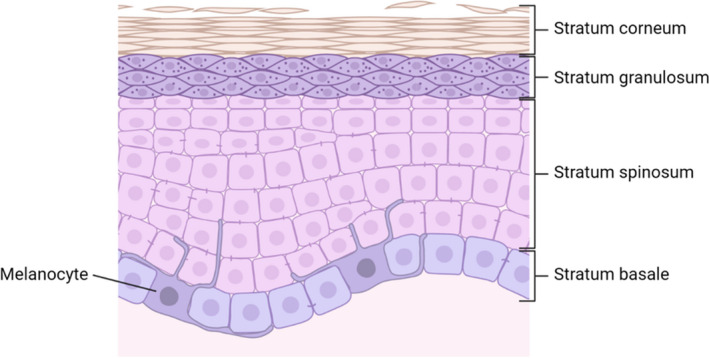
Illustration of the human epidermis. Note the desquamating sections of the stratum corneum and the melanin‐producing melanocyte cells in the stratum basale. Made with BioRender.com

In contrast to melanoidins, natural pigmentation occurs in the deeper layers of the skin.^30^ The role of pigmentation is primarily to protect from solar irradiation. Epidermal melanin (Figure 5), the main component of skin pigmentation, absorbs and dissipates a substantial portion of solar UV through its highly conjugated electronic structure. Constitutive skin pigmentation is the result of genetically determined epidermal melanin levels, although there are regulatory factors that affect total epidermal melanin levels. The most commonly known regulatory factor affecting melanin levels is exposure to UV, or solar tanning. Solar tanning is achieved through the mechanism of melanogenesis, which occurs in the melanocyte cells in the deeper stratum basale (Figure 6).^30^

### 2.3 The dangers of ultraviolet radiation

The dangers of overexposure to solar UV have been well documented. The World Health Organization even referring to the “global burden of disease from solar ultraviolet radiation.”^31^ Of the many deleterious effects of solar UV, the most noteworthy are the increased risk of skin cancers, premature ageing, extreme damage to the human eye and commonly causes skin burns.^31^, ^32^ Skin cancer is the most common form of cancer, accounting for over 40% of cancer cases.^33^ There are three main types of skin cancer; basal cell skin cancer, squamous cell skin cancer, and melanoma. It is estimated that approximately 93% of skin cancer cases are caused by exposure to UV, which increases the risk of all three main skin cancer types.^34^ The UV‐induced increased cancer risk appear to be caused primarily by the generation of free radicals and the formation of reactive oxygen species (ROS). Similarly, photoaging is caused by the formation of free radicals (both oxygen and nitrogen containing species) as a result of UV‐induced inflammation, depletion of cellular antioxidants, and antioxidant enzymes, and DNA damage.

**FIGURE 7:**
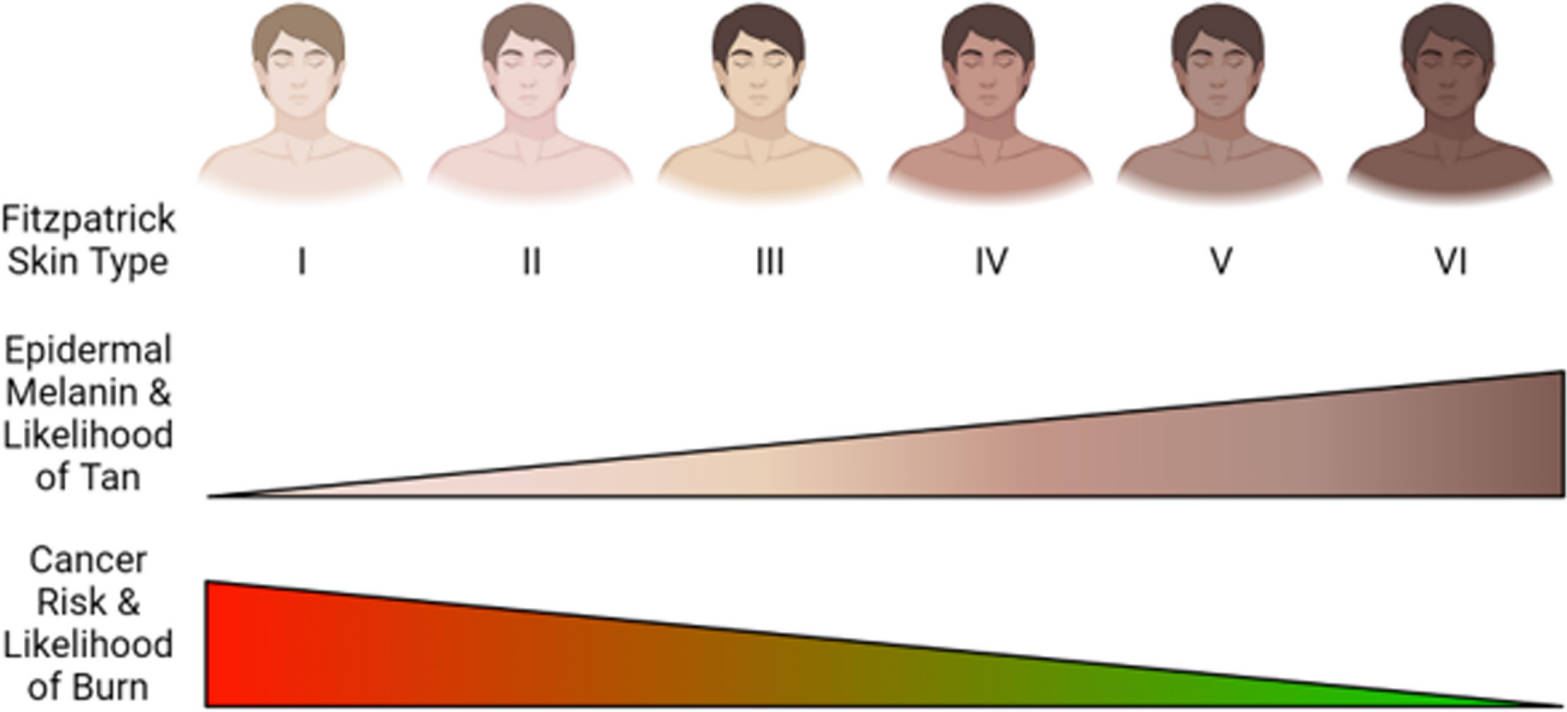
Representations of the Fitzpatrick skin types and the factors associated with their classification. For example, skin type I has low epidermal melanin and low tanning response, but a high cancer risk and chance of burning under UV exposure. Made with BioRender.com

It is generally agreed that the dangers of ultraviolet radiation vary with skin tone or skin‐type. “Skin‐typing” is typically evaluated using the Fitzpatrick scale, where six different skin phototypes (I–VI) are described according to erythema and tanning response to UV radiation^35^ with higher numbers associated with darker tones and comparatively lower burn risk (Figure 7).

The dangers of solar UV can be mitigated through several different ways. Examples include limiting exposure by remaining indoors, especially during the intense midday sun, and the use of clothing. These strategies are effective but often run counter to the sun‐seeking behaviors displayed by individuals that desire a tanned appearance. Another popular method of mitigating solar UV exposure is the use of topically applied sunscreens, which either chemically or physically block some or all of the harmful UV. The actual radiation blocked by the sunscreen is dependent on the UV blocking ingredient(s) used. The performance of sunscreens is quantified by its sun‐protection factor (SPF), measuring the fraction of UVB radiation with approximate wavelengths of 280–315 nm that can reach the skin. There are several other rating systems used by sunscreens to quantify their protection to the UVA wavelengths of 315–400 nm, such as SPF equivalence or the star rating system. The stated values on a product, however, are based on sufficient volume being applied (and reapplied), and there are often problem areas such as around the eyes that are regularly missed, and products such as moisturizers are rarely applied at sufficient quantities to achieve full protection.^36^, ^37^

There is debate as to whether the melanoidins produced as part of DHA application provides any sun protection.^38^, ^39^, ^40^ Although many sunless tanning products report an SPF rating and contain sun protecting ingredients, these benefits are only present while the product is present on the skin and not after washing or excessive perspiration. Sunless tanners must be made more aware that they are still susceptible to sunburn and other UV overexposure side‐effects despite their tanned appearance, and should therefore still use appropriate sunscreen and engage in other UV‐protective behaviors prior to extended UV exposure.^41^, ^42^

It is not possible to directly compare the UV‐associated skin cancer risks for people who have used sunless tanning products as there are no epidemiological studies on this topic. However, the safety profile of DHA is well documented and the 2010 Scientific Committee on Consumer Safety found that there was “…no reason to consider DHA as an in vivo mutagenic/genotoxic substance.” In addition, that DHA used “…as a self‐tanning ingredient in cosmetic formulations up to 10% will not pose a risk to the health of the consumer.” As such, for those who seek a tan, DHA represents a considerably safer choice compared with UV‐induced tanning.^43^

## 3 DHA SUNLESS TANNING

### 3.1 DHA sunless tanners in comparison to UV tanning

Beyond their comparative safety compared with UV‐tans, DHA sunless tanning products have several advantages. Sunless tanners do not rely on solar exposure, and are available year‐round for a consistent tan appearance. Color depth can be controlled, either through duration of application or through multiple applications. Indeed, although ostensibly a negative, the transient nature of the tan is considered advantageous for many users as it provides greater control over their appearance. Many sunless tanning products are multifunctional and contain other daily or regular skincare products such as moisturizers and hydrators, and can be added as part of a regular skincare routine.

Unfortunately, there are negatives associated with DHA sunless tanners; primarily related to user error and problems inherent to the DHA reaction. User error issues can be caused by insufficient preparation, the lack of a dedicated applicator, and partial or incomplete skin coverage. These can manifest as an uneven or streaky tan and can in part be alleviated through provision of adequate advice supplied with the product. The fundamental DHA issues are more challenging. Stronger reaction areas are obtained where the SC is thicker, such as hands, feet, and elbows. There are also different response rates between individuals, with some users responding more or less strongly to the tanning reaction.^44^ These issues are comparatively minor; however, a much bigger challenge is obtaining a natural looking tan tone.

**FIGURE 8:**
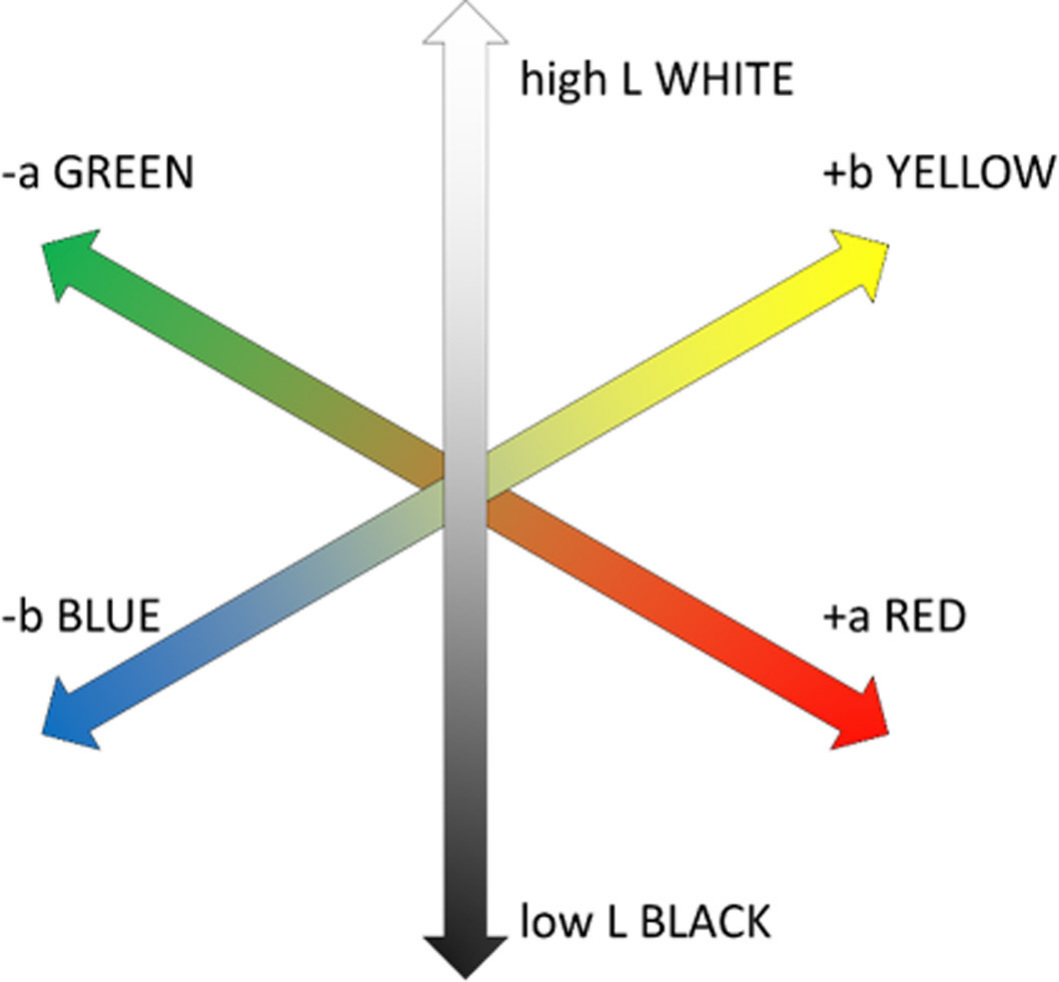
CIELAB axes of color

To quantify the comparison between UV tans and sunless tanning product tans the Commission Internationale de l'Éclairage (CIE, the International Commission on Illumination) L*a*b* color space is used, known as CIELAB (Figure 8). CIELAB space was designed to correlate to visibly perceptible color changes by the human eye. L* is a measure of lightness, with a value of 0 being pure black and 100 being pure white. The a* parameter measures along an axis running from negative scores in green spectrum to positive scores in the red, whereas the b* parameter measures along an axis from blue in the negative and to yellow in the positive.

Several research outputs have been produced to characterize the color change in skin after DHA or UV‐induced tanning.^45^, ^46^, ^47^ Typical DHA quantities in sunless tanners is between 4% and 8%, although slightly lower or higher concentrations can be used in either gradual tanners or fast‐acting/dark tanners.^48^ For color changes arising from low DHA exposure, the resulting tan remains similar to a UV‐induced tan as the changes in L*, a*, and b* are small. As DHA concentrations are increased and the tanning effect becomes more pronounced, the color profile of the sunless tan often moves more strongly toward a yellow coloration (increased b in CIELAB) when compared with a similar UV‐induced tan.^47^, ^49^ This increased contribution of yellow to the overall tan color is likely the culprit for the unnatural tan stereotype that sunless tanning products gained in the late 20th century and by a recent US president.^50^

### 3.2 Formulation considerations and limitations

DHA is very reactive that presents limitations when formulating a DHA‐based sunless tanning product.^48^ The DHA has a limited shelf‐life and requires storage in cool, dry environments to minimize degradation. All primary and secondary amines, including amino acids, are considered incompatible chemicals in a DHA sunless tanner formulation as they will rapidly react with the DHA. This is also true of most other nitrogen‐containing compounds, including small quantities that may be present as precursor or raw material impurities in some purchased compounds such as taurate‐based thickeners. Oxidizing agents should also be considered incompatible with DHA. Care must also be taken to ensure the absence of metals, especially iron, copper, manganese, and titanium, because these also behave as oxidizing agents for DHA.^9^ DHA is also relatively heat instable and therefore needs to be added during the final cool down process steps of formulation.^9^

Once in solution, DHA exhibits a tendency to acidify the solution pH over time,^9^ dropping a freshly prepared solution of pH 4–5.5 to around pH 3 due to the formation of organic acids. The starting pH of a solution is a compromise between ensuring the stability and longevity of the DHA, while ensuring a safe and irritation‐free product when applied to the skin. pH is controlled through buffering solutions using a conjugate acid/base pair without an amine moiety, such as acetic acid/acetate, citric acid/citrate, or a phosphate buffer. All other chemicals in the formulation must be stable and able to perform their respective roles at the low pH expected of a DHA sunless tanning product.

### 3.3 Different product types

**FIGURE 9:**
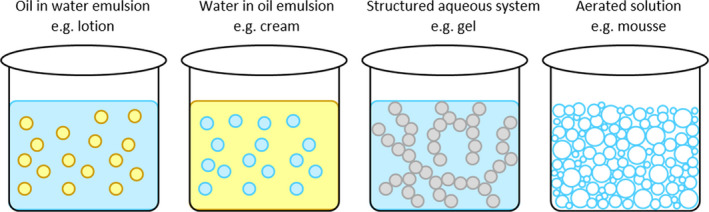
Simple model of different product types, from left to right: oil in water emulsion, water in oil emulsion, structured water‐based gel, water‐based mousse (oil shown in yellow, water shown in blue, structuring agent shown in gray, and bubbles shown in white)

DHA is compatible with oil in water, and water in oil emulsions, along with aqueous gels and mousses.^9^ This has resulted in multiple product types, many of which alleviate different issues associated with DHA sunless tanners (Figure 9). Creams and lotions are the most common product type, typically more or less viscous water in oil emulsions, respectively. Both are easy to use, alleviating application issues such as streaks and are also compatible with most cosmeceutical additives such as emollients and hydrating agents. Gel delivery vehicles are more difficult to formulate as most commercially available thickening agents including carbomers and magnesium aluminum silicate are incompatible with DHA. There are, however, some thickening agents that are compatible with DHA (such as hydroxymethylcellulose and methylcellulose), and so, a few gel formulations exist in the market. Gelées (water‐in‐silicone emulsions) can also be formulated, using silicones such as dimethicone and cyclomethicones.^48^ DHA mousses and foams, are often consumer favorites due to their relatively fast drying times and compatibility with oilier skin types. These advantages are a result of the high‐water content of mousses and foams. Due to the fast drying times of the products, mousses and foams typically contain a guide color. This is a colored pigment contained in the formulation, designed to be representative of the end‐color that enables the user to see any areas where they have not yet applied the product to. The most recent sunless tanning product type is the mist, or spray. These water‐based solutions tend to contain a low concentration of DHA and are suitable for light applications to the face.

## 4 IMPLICATIONS OF SUNLESS TANNING

### 4.1 Trends in sunless tanning

Several surveys with a large number of participants have been conducted in recent years, to determine the demographics of the tanning population and their sun exposure behaviors. These studies have shown that a greater proportion of users of sunless tanning are female, although this varies by location, additionally the values for the percentage of female users have ranged between 63% and 95%.^41^, ^51^ Fitzpatrick skin types I, II, and III are more likely to use tanning products,^51^ while race/ethnicity also appears to be relevant with one study reporting non‐Hispanic whites to have the highest weighted prevalence of 8.6%, decreasing to 3.0% for Hispanics, 1.0% for non‐Hispanic Asians, and finally 0.6% for non‐Hispanic blacks.^52^ Several authors report an increased prevalence of sun burns in the tanning product users in their surveys.^51^, ^52^, ^53^ This trend is sometimes explained by the lack of education or clear advertising that a sunless tan does not give the same level of UV protection as a UV‐induced tan, although it is also likely a result of tan‐seeking behaviors that tanning product users also display.^51^, ^52^, ^53^, ^54^ Interestingly, some studies found an increased use of sunscreen among their sunless tanning product using respondents.^54^ Some other interesting factors that have been associated with tanning product use includes working part‐time,^41^ being college educated, and being non‐obese.^52^

### 4.2 Advising the consumer

Improper preparation or use of the sunless tanning product by the consumer can lead to a poor end‐result, which can also lead to poor publicity for the individual^50^ and the business. A strong emphasis should be given to advising consumers including giving clear and concize directions for use. A common source of advice for the modern consumer has also become online and social media influencers.^55^, ^56^

Current advice for topical application of sunless tanning products is focused on the preparation of the SC substrate, topical application, and development time. The skin should be in a healthy condition. Adequate hydration and moisturization should be performed at least the day before the application of the sunless tanning product.^48^ An even substrate is required for optimal tan appearance and longevity; encouraging consumers to exfoliate before application is recommended.^48^ Similarly, the use of a dedicated glove can help achieve even product distribution while simultaneously protecting the hand. This is important as the hands are an area of customer dissatisfaction due to the increased expression of the tan appearance. The hands should be tanned for a shorter duration with the same product, or with an alternative product with a lower DHA concentration. A lighter application of a facial mist is recommended when attempting to tan the face. To reduce the risk of smudges or streaks resulting from friction or perspiration, loose‐fitting clothes should be worn and activity should be kept to a minimum. For best results, it is advised to apply sunless tanning products in the late‐evening after exfoliating in the shower; so that, the user can then wear loose‐fitting pyjamas and engage in minimal movement during sleep.

### 4.3 Potential health risks and benefits

As the popularity of sunless tanning products has increased, the examination of their potential health risks and benefits has also grown (as extensively reviewed recently).^4^, ^5^, ^6^, ^57^ The clear and major benefit from DHA sunless tanning products is the reduction of UV exposure. In addition, a tanned appearance is strongly related to a general sense of emotional and physical wellbeing, physical attractiveness, and improved body image.^58^ A more tanned appearance may also provide visual cues to human health.^59^ There have also been many reported beneficial effects of melanoidins and Maillard reaction products to human physiology in terms of antioxidant, antimicrobial, and prebiotic activities.^60^ Although these have only been examined in the context of dietary intake, any antioxidant and antimicrobial activities are likely to be of some advantage in the SC. An unresolved safety concern of topically applied DHA is whether it may penetrate deeper than the SC.^61^ Our own experimental data indicate that this is unlikely when an effective barrier is present. Unsurprisingly, dry skin and eventually skin irritation by avid sunless tan users that engage in excessive applications of the product has been reported, as has contact dermatitis in an experimental model using Mexican hairless dogs overexposed to topically applied solutions of DHA.^62^ Again, these points emphasize the importance of clear messaging and consumer education on general skin health in addition to product specific directions.

## 5 CONCLUSION

Although there are some safety considerations around DHA and its use in tanning products, it remains a safer alternative to the deleterious effects of UV overexposure. DHA remains the main tanning active despite its many formulation limitations. The mechanism of tanning, the Maillard reaction, remains incompletely characterized and would be a valuable topic for further investigation. Advances in its understanding could result in optimization and improvement of the sunless tanning end‐product. Greater insights into the activity of DHA after topical application to the stratum corneum could also be obtained, particularly with respect to its true penetration depth and other safety concerns. In conclusion, DHA sunless tanning offers an attractive route to a UV‐free tan for the consumer, a complex reaction mechanism for chemists and biologists to further explore, and poses some unique challenges for the business formulator.

## CONFLICT OF INTEREST

The authors acknowledge the potential conflict of interest by working with/for PZ Cussons (International) Limited, but maintain that it did not prejudice the impartiality of the research reported.

## AUTHOR CONTRIBUTIONS

Josh Turner wrote the draft of the article. Danielle A. O’Loughlin prepared the figures and edited the draft. Phill Green, Thomas O. McDonald, and Kevin J. Hamill edited and wrote the final version of the manuscript.

## ETHICAL APPROVAL

The authors declare that no ethics approval was needed for this literature review.

## Data Availability

Data sharing not applicable to this article as no datasets were generated or analysed during the current study.
